# Pre-exposure prophylaxis service among men who have sex with men in Malaysia: findings from a discrete choice experiment

**DOI:** 10.1038/s41598-023-41264-5

**Published:** 2023-08-30

**Authors:** Alex Dubov, Frederick L. Altice, José I. Gutierrez, Jeffrey A. Wickersham, Iskandar Azwa, Adeeba Kamarulzaman, Kamal Gautam, Roman Shrestha

**Affiliations:** 1 School of Behavioral Health, Loma Linda University, Loma Linda, CA USA; 2 Department of Internal Medicine, Section of Infectious Diseases, Yale School of Medicine, New Haven, CT USA; 3 Centre of Excellence for Research in AIDS (CERiA), Faculty of Medicine, University of Malaya, Kuala Lumpur, Malaysia; 4 Department of Family Health Care Nursing, School of Nursing, University of California, San Francisco, San Francisco, CA USA; 5 Infectious Diseases Unit, Department of Medicine, Faculty of Medicine, University of Malaya, Kuala Lumpur, Malaysia; 6 Department of Allied Health Sciences, University of Connecticut, 358 Mansfield Rd, Unit 1101, Storrs, CT 06269 USA

**Keywords:** Health care, Health services

## Abstract

Men who have sex with men (MSM) in Malaysia are disproportionately affected by HIV. As pre-exposure prophylaxis (PrEP) is being introduced, we assessed population-based PrEP delivery preferences among MSM in Malaysia. We conducted a discrete choice experiment through an online survey among 718 MSM. The survey included 14 choice tasks presenting experimentally varied combinations of five attributes related to PrEP delivery (i.e., cost, dosing strategy, clinician interaction strategy, dispensing venue, and burden of visits to start PrEP). We used latent class analysis and Hierarchical Bayesian modeling to generate the relative importance of each attribute and preference across six possible PrEP delivery programs. PrEP dosing, followed by cost, was the most important attribute. The participants were clustered into five preference groups. Two groups (n = 290) most commonly preferred on-demand, while the other three preferred injectable PrEP. One group (n = 188) almost exclusively considered cost in their decision-making, and the smallest group (n = 86) was substantially less interested in PrEP for reasons unrelated to access. In simulated scenarios, PrEP initiation rates varied by the type of program available to 55·0% of MSM. Successful PrEP uptake among Malaysian MSM requires expanding beyond daily oral PrEP to on-demand and long-acting injectable PrEP, especially at affordable cost.

## Introduction

The HIV epidemic in the Association of Southeast Asia Nations (ASEAN) region is concentrated among key populations, with three-quarters of new infections occurring among MSM, people who use drugs, transgender women, and sex workers^1^. The highest share of new infections is among MSM (~ 60%)^2^, as the risk of acquiring HIV in MSM is 27 times higher than in other key populations^3^. Pre-Exposure Prophylaxis (PrEP) has been found to be an effective biomedical intervention for HIV prevention. The World Health Organization (WHO) recommends PrEP for people at risk of HIV infection, such as MSM^4^. Yet only 25% of MSM in the region are covered by any HIV prevention services, while PrEP is mostly available in small pilot trials^5^. According to evidence, health services that match individual treatment preferences positively affect treatment uptake and outcomes^6^. Several factors contribute to the optimal design and implementation of a PrEP program, including how MSM access the program, how they take the medications, their costs in terms of money and convenience, and the clinical burden on MSM in order to receive the medication^7^. Individual health beliefs may also contribute to preferences in service delivery. Therefore, PrEP programs that are tailored to address the unique needs and challenges of MSM are urgently needed for the rapid scale-up of PrEP globally.

In Malaysia, a low-to-middle-income country (LMIC), HIV cases are rising among MSM, from 19% in 2012 to 62% in 2021, with MSM expected to account for three-quarters of all cases by 2030^8^. Malaysia’s Ministry of Health Malaysia prioritizes PrEP as part of the National Strategic Plan to end AIDS by 2030^9^ yet will licensure for HIV prevention is new, and the government intends to subsidize it starting in 2023^10^. As Malaysia emerges with a pilot PrEP program with only ~ 300 MSM on PrEP, the PrEP implementation gap is extraordinary to meet the needs of the estimated 220,000 MSM who can benefit from it. With high levels of stated interest in and awareness about PrEP^11^, government subsidy will only play one part of the role in optimal scale-up, especially in a country like Malaysia where there are extraordinary levels of stigma and frank discrimination toward MSM^12^ as same-sex behavior is criminalized in secular and Shariah law^13^ and reinforced in healthcare settings^14^. Innovations in addressing the complex interplay between individual and healthcare provider stigma, along with costs and other concerns related to PrEP, are crucial to understanding in order to develop PrEP delivery programs that are meaningful for MSM at risk for HIV.

As Malaysia moves to approve and support PrEP for HIV prevention, it is crucial to enhance our understanding of preferences among MSM in how PrEP delivery will meet their needs to properly guide implementation and scale-up. Optimal scale-up of PrEP is most likely to succeed when the preferences of MSM are incorporated into the way it is delivered. General considerations expressed by MSM in Malaysia about what is most important to them include the type of medication they take, how they take it, out-of-pocket expenses, where they get it, and the clinical burden of getting started^15^. Stated preference methods, such as conjoint analysis, are powerful tools to quantify end users’ preferences for new programs, including PrEP^16^. The conjoint analysis assumes that MSMs consider the PrEP program as a composition of attributes (e.g., price, delivery method) and will place a certain amount of value (part-worth utility score) on each attribute. The objective of this study was to evaluate the preferences of the Malaysian MSM on specific attributes related to PrEP programs using conjoint analysis. Segmentation analysis was further used to examine whether and how MSM’s preferences cluster into “phenotype” segments to guide optimal PrEP delivery model. This “patient-centered” approach recognizes the needs of end-users (i.e., MSM) and the context within which they live to inform implementation strategies to improve the scale-up of PrEP, which can be used in Malaysia and extended to other ASEAN regions.

## Methods

In August–September 2021, we recruited a convenience sample of Malaysian MSM to complete a cross-sectional anonymous online survey. Inclusion criteria were: (1) being 18 years or older; (2) identifying as cis-gender male; (3) reporting a history of sex with another male; (4) reporting HIV negative status or HIV status unknown; and (5) being able to read and understand English.

Participants were recruited online using the following methods: (1) paid, targeted banner ads on social networking sites (e.g., Facebook); (2) flyers on pages of non-governmental organizations (NGOs) and community-based organizations (CBOs) that provide services to MSM, and (3) direct advertisement on geosocial networking (GSN) apps used by MSM (e.g., Hornet). Those interested could click on a link to access an online study disclosure form with a ‘Click to consent’ procedure. All participants provided informed consent to participate in the survey after reading the potential risks and benefits of the study and clicking ‘Click to consent’ before proceeding with the online survey. On average, participants took 15–20 min to complete the survey and were not compensated financially. The study was approved by the University of Malaya Research Ethics Committee (UMREC) and the University of Connecticut Institutional Review Board. All procedures involving human participants were conducted in accordance with ethical standards and the Helsinki Declaration of 1964.

A choice-based conjoint (CBC) survey to assess preferences for PrEP delivery programs was developed based on a review of the literature and in-depth discussions with Malaysian stakeholders. We included five different PrEP delivery program attributes: dosing method (daily oral tablet, on-demand tablet regimen, injection every two months); cost (RM 125–249 and RM 250–350); clinician interaction strategy (in-person, online, or text-based consultation); number of visits to start PrEP (same day, one week); and dispensing venue (private courier, mail delivery, self pick-up). Combined costs for PrEP may include costs for the medication and secondary costs for STI testing. The survey design prohibited infeasible attribute combinations, such as injectable PrEP with mail delivery, to ensure that the choices presented to participants aligned with realistic and plausible scenarios. This enhanced the validity and relevance of the study results. The survey was piloted with ten MSM to ensure attribute levels were understood, relevant, and logical when combined to create a PrEP program.

Additionally, we collected the following data: (1) demographic characteristics: age, sexual orientation, ethnicity, educational and relationship status, and income; (2) psychosocial factors: participants were asked about their experience of depressive symptoms in the past two weeks using the PHQ-2 scale, a two-item screening tool. The scores range from 0 to 6, with a score of 3 or higher indicating the presence of major depressive disorder^17^. We also asked participants two questions about stigma and acceptance of their sexual orientation: “Have you told your family about your sexual orientation?” (‘*No’; ‘Yes, but only a few family members know’; ‘Yes, most of my family members know’*) and “Has a healthcare provider ever discriminated against you because of your sexual orientation? For example, treating you unfairly or denying you care/treatment”; (3) recent sexual behavior and substance use (in the past 6 months): engagement in anal sex with another man, the number of sexual partners, HIV status of their sexual partner, receiving a diagnosis for a sexually transmitted infection (e.g., Chlamydia, Gonorrhea, Syphilis), involvement in transaction sex and chemsex (i.e., use of psychoactive drugs before or during sex)^18,19^, and using of substances (e.g., ecstasy, methamphetamine, ketamine). We also asked participants if they had tested for HIV and injected drugs; (4) perceived HIV risk: participants were asked to self-assess their perceived risk of contracting HIV in the following 6 months using a four-point rating scale (*‘not likely at all’*; *‘a little likely’*; *‘somewhat likely’*; *‘extremely likely’*); and (5) PrEP knowledge and history: participants were asked whether they had ever heard of PrEP before this survey and if they had ever used PrEP. If participants indicated having used PrEP, they were then asked if they were currently using PrEP. Participants were also asked if they were willing to use (or continue using) PrEP to reduce their risk of contracting HIV, how much they were willing to pay for PrEP, awareness about injectable PrEP, and their preference for PrEP modality (i.e., daily, on-demand, and injectable). In addition, six-item questionnaires related to concerns related to injectable PrEP for HIV prevention were asked^20^.

Preferences for hypothetical PrEP programs were solicited by asking participants to complete 14 choice tasks presenting experimentally varied combinations of the study's attribute levels^21^. These CBC tasks required participants to make a series of trade-offs between the levels of the study’s five attributes. Each CBC exercise included a ‘None’ option, representing the likelihood of a participant selecting no PrEP program option versus the hypothetical PrEP program offered within the exercise. Sawtooth Software’s experimental design module generated 650 different versions of the survey according to three principles: (1) each attribute level appeared only once in each choice task; (2) across choice tasks, each attribute level appeared as close to an equal number of times as possible; and (3) attribute levels were chosen independently of each other^22^. All 650 unique survey versions were used. The remaining 68 respondents received the same design as the previous 650, which poses no harm given hundreds of unique versions in the total design pool. The cognitive burden of the survey was minimized through the use of choice tasks, which simulate familiar, everyday choices. The number of tasks (14) is standard practice for CBC surveys, and the pilot test showed that participants were able to complete the survey without difficulty. The standard error for each level was 0·03, and the efficiencies reported were all 1·000. We used Johnson and Omre’s formula to determine that a sample size of 150 or more is necessary to yield reasonably precise estimates of utility levels in a five-attribute, three-level, two-choice model.

Utility scores were then used to predict the share of preference (participation interest) among hypothetical PrEP program scenarios. PrEP program scenarios were configured to represent each group’s highest share of preference (or participation interest). For this study, participation rates for these PrEP scenarios were generated using the randomized first-choice model. The randomized choice model accounts for variation in each participant's total utility for each option and error in point estimates of the utility and has been shown to have better predictive ability than other shares of preference models^23^.

## Analysis

To obtain a consistent sample, we eliminated entries that took less than 10 min, as the pilot testing required at least 10 min to complete the survey. In addition, security features within the Sawtooth software eliminated responses from the same internet protocol (IP) address. Additional security measures included captcha verification and validation items to identify inconsistent or unreliable responses. In total, 1286 individuals opened the survey, and 1041 (80·9%) met the inclusion criteria. Of the 1041 respondents who started the survey, 323 (31·0%) entries did not meet data quality parameters (e.g., incomplete data entry, the same answer for all items, survey time under 10 min) and were excluded, thus leaving the final analytic sample to 718.

Data analysis began with generating descriptive statistics of the aggregate sample, including frequencies and measures of central tendency. The Hierarchical Bayes (HB) model was used for conjoint data to estimate part-worth utility scores (PWUS) across all 13 attribute levels. The resulting PWUS of the levels are zero-centered, meaning that scores further away from zero indicate a more substantial positive or negative preference for an attribute level when compared to the other level choices under the same attribute. The mean attribute relative importance scores (RIS) were also generated using Hierarchical Bayes (HB) analysis. They were rescaled to 100 to characterize the magnitude of influence each attribute has on the respondents' decision-making process. Latent class multinomial logit analysis was used to segment participants into groups with similar preferences, and we chose a 5-group solution based on the consistent Akaike Information Criterion (AIC).

We used the Sawtooth Lighthouse Studio 9·12·1 market simulation module to estimate preferences separately for six hypothetical PrEP programs versus no PrEP (None). The programs are described in Table 1. Using the market simulation module, we predicted preferences for hypothetical PrEP programs based on the utilities obtained during the conjoint analysis. Program interest rates for these PrEP scenarios were generated utilizing the randomized first-choice (RFC) model. This model assumes that participants prefer the option with the highest utility. While the RFC model is better suited for simulations with latent class models, simulating on the Hierarchical Bayes draws can sometimes produce slightly superior results. Using this model, we were able to predict the percentage of participants in each group who would choose a hypothetical PrEP program (i.e., PrEP participation rates). This approach assumes that each participant chooses the option with the highest composite utility, adjusting for both attribute and program variability.

**Table 1:** Description of hypothetical PrEP scenarios with different attributes and levels^a^.

PrEP scenario	PrEP attributes & level options				
	Dosing method	Cost	Clinician interaction strategy	Dispensing venue	Burden of visits to start PrEP
Injectable best case	Injectable	125–249	In-person consult	Self pick-up	One week wait
Injectable worst case	Injectable	250–350	In-person consult	Self pick-up	One week wait
Oral PrEP best case	Daily oral	125–249	Online consult	Private courier	Same day
Oral PrEP worst case	Daily oral	250–350	In-person consult	Self pick-up	One week wait
On-demand best case	On-demand	125–249	Online consult	Private courier	Same day
On-demand worst case	On-demand	250–350	In-person consult	Self pick-up	One week wait

^a^Scenarios descriptions reference attribute characteristics listed in Tables 2 and 3.

## Results

### Participants

The characteristics of the 718 participants, stratified by participant segmentation group, are described in Table 2 and Table 3. Participants were generally in their early 30 s (mean = 31 years, standard deviation = 8 years). Over half (51·5%) were Chinese, followed by Malay (28·8%). Three-quarters of the sample (74·0%) identified as gay, single (70·6%), and not out to their families (67·6%). Most were university graduates (69·5%) with an average monthly income of $1115. One-third of participants (31·5%) had never tested for HIV, and 11·3% had a recent STI diagnosis. Additionally, 39·9% reported that they perceived themselves as being at risk of acquiring HIV. Similarly, 60·3% used condoms inconsistently, with 20·6% never using condoms, while 10·3% had partners who live with HIV. A conservative estimate based on several reported HIV risk factors suggests that at least 30% to 40% of the sample met the eligibility criteria for PrEP.

**Table 2:** Socio-demographic characteristics of the participants (N = 718).

Variables	Total	Group 1	Group 2	Group 3	Group 4	Group 5
		“High risk, LAI PrEP”	“At risk, affordable PrEP”	“High risk, no on-demand PrEP”	“Low risk, no PrEP”	“High risk, on demand PrEP”
	N = 718	n = 125	n = 188	n = 115	n = 86	n = 204
Age	31·8 (8)	31·8 (8)	32·3 (9)	32·7 (9)	30·6 (8)	31·3 (8)
Sexual orientation						
Gay	532 (74·0)	89 (71·2)	140 (74·4)	91 (79·1)	61 (70·9)	151 (74·0)
Straight	147 (20·0)	32 (25·6)	39 (20·7)	20 (17·3)	14 (16·2)	42 (20·5)
Bisexual	7 (1·0)	1 (0·8)	4 (2·1)	0	1 (1·1)	1 (0·5)
Queer	19 (2·6)	1 (1·6)	2 (1·0)	2 (1·7)	7 (8·1)	6 (2·9)
Other	13 (1·8)	1 (0·8)	3 (1·6)	2 (1·7)	3 (3·4)	4 (1·9)
Ethnicity						
Chinese	370 (51·5)	69 (55·2)	99 (52·6)	58 (50·4)	45 (52·3)	99 (48·5)
Indian	42 (5·8)	6 (4·8)	17 (9·0)	4 (3·4)	6 (6·9)	9 (4·4)
Malay	207 (28·8)	34 (27·2)	47 (25·0)	32 (27·8)	27 (31·3)	67 (32·8)
Mixed	31 (4·3)	2 (1·6)	7 (3·7)	7 (6·0)	5 (5·8)	10 (4·9)
Native sabahan	18 (2·5)	2 (1·6)	5 (2·6)	2 (1·7)	2 (2·3)	7 (3·4)
Native sarawakian	30 (4·1)	7 (5·6)	8 (4·2)	8 (6·9)	1 (1·1)	6 (2·9)
Other	20 (3·0)	5 (4·0)	5 (2·6)	4 (3·4)	0	6 (2·8)
Education						
Primary school	2 (0·2)	0	0	0	0	2 (0·9)
Secondary school	60 (8·1)	7 (5·6)	14 (7·3)	11 (9·5)	11 (12·7)	17 (8·2)
Pre-university	157 (21·8)	24 (19·2)	47 (25·0)	22 (19·1)	14 (16·2)	50 (24·5)
Post-graduate	499 (69·5)	94 (75·2)	127 (67·5)	82 (71·3)	61 (70·9)	135 (66·1)
Average monthly income (RM)	4936	5339	4724	5691	4585	4453
Relationship status						
Single	507 (70·6)	82 (65·6)	147 (78·1)	77 (66·9)	58 (67·4)	143 (70·1)
Married/partnered	203 (28·2)	43 (34·4)	39 (20·7)	37 (32·1)	28 (32·6)	56 (27·4)
Divorced/widowed	8 (1·1)	0	2 (1·0)	1 (0·8)	0	5 (2·4)
PHQ-2						
< 3	247 (34·5)	47 (37·6)	60 (32·0)	39 (34·0)	24 (27·9)	77 (37·8)
3 or greater	471 (65·5)	78 (62·4)	128 (68·0)	76 (66·0)	62 (72·1)	127 (62·2)
Came out to family						
No	486 (67·6)	79 (63·2)	132 (70·2)	71 (61·7)	58 (67·4)	146 (71·5)
Yes, a few know	154 (21·4)	34 (27·2)	35 (18·6)	32 (27·8)	17 (19·7)	36 (17·6)
Yes, most know	78 (10·8)	12 (9·6)	21 (11·2)	12 (10·4)	11 (12·7)	22 (10·7)
Healthcare discrimination	91 (12·6)	16 (12·8)	30 (15·9)	11 (9·5)	7 (8·1)	27 (13·2)

**Table 3:** Sexual behaviors and drug use among participants.

Variables	Total	Group 1	Group 2	Group 3	Group 4	Group 5
		“High risk, LAI PrEP”	“At risk, affordable PrEP”	“High risk, no on-demand PrEP”	“Low risk, no PrEP”	“High risk, on demand PrEP”
	N = 718	n = 125	n = 188	n = 115	n = 86	n = 204
Anal sex, past 6 months (Yes)	460 (64·0)	88 (70·4)	129 (68·6)	83 (72·1)	41 (47·6)	119 (58·3)
Condomless sex	277 (60·2)	55 (62.5)	80 (62·0)	53 (63·9)	24 (58.5)	65 (54·6)
Paid to have sex	34 (7·4)	6 (4·8)	12 (6·3)	4 (3·4)	2 (2·3)	10 (4·9)
HIV + partners	72 (10·3)	15 (12·0)	14 (7·4)	18 (15·6)	3 (3·4)	22 (10·7)
Substance use, past 6 months						
Ecstasy	11 (1·5)	0	3 (1·5)	2 (1·7)	2 (2·3)	4 (1·9)
Methamphetamine	57 (7·9)	14 (11·2)	16 (8·5)	11 (9·5)	4 (4·6)	12 (5·8)
Ketamine	5 (0·6)	0	2 (1·0)	1 (0·8)	1 (1·1)	1 (0·4)
GBH	25 (3·4)	5 (4·0)	10 (5·3)	6 (5·2)	0	4 (1·9)
Chem sex	61 (8·5)	16 (12·8)	16 (8·5)	12 (10·4)	2 (2·3)	15 (7·3)
Injected drugs	19 (2·6)	6 (4·8)	5 (2·6)	2 (1·7)	1 (1·1)	5 (2·4)
HIV test						
Never	226 (31·5)	35 (28·0)	56 (29·8)	30 (26·1)	30 (35·0)	75 (36·8)
> 1 year ago	330 (46·0)	58 (46·5)	94 (50·0)	65 (56·5)	26 (30·0)	87 (43·2)
< 1 year ago	162 (22·5)	32 (25·5)	38 (21·2)	20 (17·4)	30 (35·0)	42 (20·5)
Any STI past 6 months	81 (11·3)	11 (8·8)	24 (12·8)	13 (11·4)	3 (3·5)	30 (14·8)
Self-perceived HIV risk						
Extremely likely	32 (4·4)	6 (4·8)	8 (4·2)	3 (2·6)	1 (1·2)	14 (6·9)
Somewhat or little likely	254 (35·4)	42 (33·6)	75 (39·9)	43 (37.4)	25 (29·1)	69 (33·8)
Not likely	432 (60·1)	77 (61·6)	105 (55·9)	69 (60·0)	60 (69·7)	121 (59·3)
Heard of PrEP	614 (85·5)	116 (92·8)	167 (88·8)	108 (93·9)	59 (68·6)	164 (80·3)
Ever used PrEP	133 (18·5)	22 (17·6)	37 (19·6)	27 (23·4)	11 (12·7)	36 (17·6)
Currently taking PrEP (n = 133)	78 (58·6)	10 (45·5)	21 (56·7)	19 (70·3)	5 (45·4)	23 (63·8)
Willing to use PrEP	667 (92·9)	116 (92·8)	184 (97·7)	112 (97·3)	65 (75·5)	190 (93·1)
Heard of injectable PrEP	66 (9·1)	13 (10·4)	13 (6·9)	14 (12·1)	8 (9·3)	18 (8·8)
Willing to use injectable PrEP	634 (88·3)	122 (97·6)	181 (96·2)	113 (98·2)	48 (55·8)	170 (83·3)
Concerns about injectable PrEP						
Fear of needles	142 (19·7)	19 (15·2)	36 (19·1)	8 (6·9)	28 (32·5)	51 (25·0)
Does not protect	225 (31·3)	41 (32·8)	56 (29·7)	47 (40·8)	22 (25·5)	59 (28·9)
Costs too much	503 (70·0)	83 (66·4)	143 (76·0)	92 (80·0)	54 (62·7)	131 (64·2)
Long-term side effects	369 (51·3)	66 (52·8)	100 (53·1)	63 (54·7)	39 (45·4	101 (49·5)
Travel to clinic	282 (39·2)	34 (27·2)	75 (39·8)	44 (38·2)	37 (43·0)	92 (45·1)
Providers coverage	282(39·2)	54 (43·2)	74 (39·3)	54 (46·9)	26 (30·2)	74 (36·2)
None	59 (8·2)	15 (12·0)	10 (5·3)	7 (6·0)	7 (8·1)	20 (9·8)
How much willing to pay for PrEP monthly						
< 200	655 (91·2)	108 (86·4)	181 (96·3)	102 (88·6)	80 (93·0)	184 (90·2)
201–400	52 (7·2)	14 (11·2)	7 (3·7)	12 (10·4)	4 (4·6)	15 (7·3)
> 400	11 (1·4)	3 (2·4)	0	1 (0·8)	2 (2·2)	5 (2·4)
Preference of PrEP modality						
Daily	160 (22·2)	4 (3·2)	44 (23·4)	23 (20·0)	21 (24·4)	68 (33·3)
On-demand	262 (36·4)	51 (40·8)	63 (33·5)	10 (8·7)	40 (46·5)	98 (48·0)
Injectable	296 (41·2)	70 (56·0)	81 (43·0)	82 (71·3)	25 (29·0)	38 (18·6)

Additionally, a majority (85.5%) of participants were aware of PrEP, yet only a few (18.5%) had ever used it, and even fewer (10.8%) were currently on PrEP. However, nearly all (92.9%) wanted to use PrEP. Awareness of injectable PrEP was low (9.1%), but the majority (88.3%) were willing to use it. Among the three available PrEP regimens, 41.2% of the sample favored injectable PrEP, followed by an on-demand regimen (36.4%) and daily oral PrEP (22.2%). The primary barriers to adopting injectable PrEP were cost (70%) and concerns regarding long-term side effects (51.3%).

### Relative importance scores and part-worth utilities

Table 4 displays the descriptions of the attributes and levels shown to the participants in the survey. Tables 5 and 6 show the relative importance scores (RIS) and the PWUS (zero-centered) of the five attributes and corresponding attribute levels stratified by the five groups. Overall, the *dosing* (RIS 38·1%) and *cost* (RIS 37·6%) were the most essential attributes among all the participants, suggesting that these attributes were the most influential factors among study participants when deciding to participate in a PrEP program. The latent class analysis generated five distinct segments. The groups differed in several characteristics.

**Table 4:** Description of conjoint survey attributes and associated level options presented to respondents.

Attributes and levels	Survey description
Cost	
RM 250–350	**RM 250–350** involves going to private clinic/hospital
RM 125–249	**RM 125–249** involves going to government clinic/hospital
Dosing	
Injectable PrEP	**Injectable PrEP** involves an injection in your arm that you receive every two months at a clinic
On-demand PrEP	**On-demand PrEP** involves taking two pills before sex one pill daily for two days after
Daily oral PrEP	**Daily oral PrEP** involves taking one pill a day
Clinician interaction strategy	
In-person	**In-person** consultation involves seeing doctor in person only
Online	**Online** consultation involves seeing doctor online only, either on your smartphone or computer
Text-based	**Text-based** consultation involves interacting with doctor using SMS/Whatsapp
Dispensing venue	
Private courier	**Private courier** involves having it delivered at your preferred location via GrabExpress, Goget, etc
Mail delivery	**Mail delivery** involves receiving it using Postal service, such as Poslaju, Fedex, or DHL
Self-pick-up	**Self-pick-up** means you will pick up PrEP yourself in the pharmacy or clinic after consultation visit
Burden of visits to start PrEP	
Same day	**Same day** after first consultation visit but provider may have to call you if there is any problem with your lab test
One week wait	**One-week wait** means waiting about a week after your first consultation visit after provider has time to review your lab results

**Table 5:** Relative importance scores (%) of PrEP program attributes by group.

Attributes	Total (N = 718) (%)	Group 1	Group 2	Group 3	Group 4	Group 5
		“High risk, LAI PrEP”	“At risk, affordable PrEP”	“High risk, no on-demand PrEP”	“Low risk, no PrEP”	“High risk, on demand PrEP”
		n = 125 (%)	n = 188 (%)	n = 115 (%)	n = 8 (%)	n = 204 (%)
Dosing	38·1	66·0	13·8	52·0	28·4	39·5
Cost	37·6	16·9	68·4	25·8	44·2	25·8
Burden of visits to start	10·4	6·2	7·7	11·7	16·4	12·1
Dispensing venue	7·6	5·9	4·1	2·5	8·0	14·4
Clinician interaction strategy	6·4	5·0	6·0	8·0	2·9	8·1

Relative importance scores reflect the influence that each attribute has on a participant’s decision-making.

**Table 6:** Part-worth utility scores (zero-centered)^a^ of PrEP program level choices by group.

Attributes	Total (N = 718)	Group 1	Group 2	Group 3	Group 4	Group 5
		“High risk, LAI PrEP”	“At risk, affordable PrEP”	“High risk, no on-demand PrEP”	“Low risk, no PrEP”	“High risk, on demand PrEP”
		n = 125	n = 188	n = 115	n = 8	n = 204
Dosing method						
Injectable PrEP	14·9	140·0	31·5	128·0	− 58·4	− 110·1
On-demand	19·6	49·0	1·4	− 131·8	80·1	78·4
Daily oral PrEP	− 34·5	− 189·0	− 32·9	3·8	− 21·7	31·7
Cost						
RM 250–350	− 94·0	− 42·3	− 171·0	− 64·6	− 110·5	− 64·5
RM 125–249	94·0	42·3	171·0	64·6	110·5	64·5
Burden of visits to start PrEP						
Same day	25·9	15·5	19·4	29·1	41·0	30·3
One week wait	− 25·9	− 15·5	− 19·4	− 29·1	− 41·0	− 30·3
Dispensing venue						
Private courier	16·3	17·1	9·2	− 3·3	16·0	33·3
Mail delivery	2·0	− 11·9	2·0	6·2	8·1	5·5
Self-pick-up	− 18·2	− 5·2	− 11·2	− 2·9	− 24·1	− 38·8
Clinician interaction strategy						
In-person	− 4·7	− 1·4	1·0	2·9	− 4·1	− 16·5
Online	16·8	13·2	14·4	18·7	9·4	23·4
Text-based	− 12·1	− 11·8	− 15·5	− 21·5	− 5·3	− 6·9
None^b^	− 388·7	− 321·3	− 388·6	− 493·7	282·4	− 653·9

^a^Zero-centered part-worth utility scores imply the positive or negative magnitude of the preference for the level choice in relation the other level options within the same attribute.

^b^“None” denotes the magnitude in which an individual is not willing to take PrEP in any scenario (i.e., a negative value in “None” represents the magnitude that an individual is willing to take PrEP in a particular scenario).

*Group 1: ‘High risk, LAI PrEP’* (N = 125, 17·4%) were almost exclusively influenced by the dosing method. Individuals in this group preferred injectable PrEP while strongly opposing the option of daily oral PrEP. They were less likely to be on PrEP, despite being the group with the highest rate of anal sex in the past six months, though with fewer partners and did so at the highest rate of sexualized drug use, including injecting drugs. Compared to other groups, members of Group 1 had a strong preference against mail delivery, potentially prioritizing privacy. Members of this group, who were more likely to identify as Chinese, straight, and married, had fewer sexual partners but reported higher chem sex engagement with these partners. They were also more likely to use and inject substances.

*Group 2: ‘At risk, affordable PrEP’* (N = 188, 26·2%) were mainly impacted by cost and held similar dosing preferences as Group 1. Members of this group were more likely to be Indian, single, and had a higher interest in PrEP. Due to their high level of perceived risk (with nearly half of the group members considering themselves at risk) and a relatively high rate of STIs, we considered members of this group as being at risk for HIV.

Cost and dosing method were the two most influential attributes for participants in *Group 3: ‘High risk, no on-demand PrEP’* (N = 115, 16·0%), with the dosing method being twice as important as cost. Members of this group strongly disliked the on-demand PrEP regimen and preferred injectable PrEP. They were slightly older with a higher average income and a higher HIV risk profile (i.e., less likely to use condoms, a higher number of HIV-positive partners, and more sex partners). However, members of this group were also more likely to have a recent HIV test; they were well-informed about PrEP and had a higher chance of using it.

MSM in *Group 4: ‘Low risk, no PrEP’* (N = 86, 12·0%) represented the smallest group but were strongly influenced by cost and preferred on-demand PrEP. They chose the ‘none’ option over 90·0% of the time, indicating their lack of interest in PrEP regardless of the program presented. Members of this group were slightly younger, with a high prevalence of depression and a lower HIV risk profile (e.g., the majority had no recent sexual contacts and a low perceived risk of HIV). This group is the least aware of PrEP.

Finally, *Group 5: ‘High risk, on-demand PrEP’* (N = 204, 28·4%), the largest group, was more heterogeneous in their preferences. Though dosing method and cost were the major contributors to preferences, unlike other groups, the dispensing venue played a more important role, including interacting directly with providers. They strongly preferred using the on-demand PrEP dosing method and the option to receive their medication by private courier. This group chose the 'none' option less than 10% of the time, indicating strong interest in PrEP and the likelihood of PrEP uptake regardless of the specific characteristic of the program. Members of this group were more likely to be Malay, less educated, and had a lower average salary. They were less likely to come out to their families. This group is the most at-risk for HIV (e.g., lowest uptake of HIV tests, higher prevalence of STI, and high perceived HIV risk).

### Preferences for PrEP programs

Preferences for PrEP across possible programs are illustrated in Table 7. We created the programs to reflect the best (e.g., cheaper same-day PrEP) and worst (e.g., more expensive PrEP after a waiting period) scenarios for each dosing method. Preferences reflect an interest in PrEP described in the preceding paragraph, with members of Groups 1 and 2 being more sensitive to changes in programs, compared to those in Groups 3 and 5, most of whom were willing to take PrEP under all scenarios. Group 4, with the least interest in PrEP, was the most sensitive to changes in the PrEP program, with participation rates varying between 9·2 and 47·1%. Overall, it is plausible to assume that PrEP initiation rates will vary by the type of program available to about 55·0% of MSM in Malaysia (groups 1, 2, and 4 are sensitive to changes in PrEP programs). In the Oral PrEP worst-case scenario, which may resemble the future roll-out of PrEP in Malaysia (more expensive daily PrEP picked up from a clinic after in-person consultation and one week wait), the overall uptake rate was 75·0%. As expected, same-day less expensive oral PrEP delivered after an online consult increased participation rates to 89%, with gains in groups 1, 2, and 4. Participation rates were slightly higher in the worst-case injectable PrEP scenario than daily PrEP (82·0% vs. 75·0%) due to gains in group 1. Both injectable and daily PrEP best-case scenarios, however, resulted in the same participation rate of 89·0%. On-demand PrEP was the most preferred scenario, with participation rates reaching 93·0% in the best-case scenario (same-day less expensive on-demand PrEP delivered after an online consult).

**Table 7:** Program interest (share of preference)^a^ rates (%) of hypothetical PrEP scenarios by group.

PrEP scenario^b^		Total population and group PrEP program interest scores					
		Total (N = 718) (%)	Group 1	Group 2	Group 3	Group 4	Group 5
			“High risk, LAI PrEP”	“At risk, affordable PrEP”	“High risk, no on-demand PrEP”	“Low risk, no PrEP”	“High risk, on demand PrEP”
			n = 125 (%)	n = 188 (%)	n = 115 (%)	n = 8 (%)	n = 204 (%)
1	Injectable PrEP best case scenario	88·9	97·9	99·7	99·9	22·2	95·3
2	Injectable PrEP worst case scenario	81·8	95·4	86·6	99·5	9·2	89·8
3	Oral daily PrEP best case scenario	89·0	81·5	99·8	99·8	37·0	99·6
4	Oral daily PrEP worst case scenario	75·1	60·3	78·3	97·6	10·7	95·8
5	On-demand PrEP best case scenario	92·9	97·2	99·9	99·0	47·1	99·8
6	On-demand PrEP worst case scenario	81·6	90·4	83·0	91·0	16·2	97·1

^a^Share of preference denotes the percent of respondents that would prefer or have an interest in the respective PrEP delivery program scenario with a particular combination of program attributes based on part-worth utilities obtained during the conjoint analysis survey.

^b^Descriptions of PrEP Scenarios 1 through 6 are presented in Table 4.

## Discussion

To our knowledge, this is the first and largest study to assess preferences for various PrEP delivery programs among Malaysian MSM, which are difficult to reach as they live in a highly stigmatized environment. Acceptability of the best possible PrEP scenario (92·9%) suggests the potential for widespread use with an optimal program design. The variability in acceptability of hypothetical programs demonstrates how attributes of PrEP programs may impact PrEP scale-up in Malaysia. Our data suggest that providing clear support for alternative dosing methods is essential when designing PrEP programs, with PrEP cost being a close second. For some, on-demand PrEP delivery may translate to lower costs unless the risk for HIV acquisition is high enough to result in daily use. While respondents varied in the importance they attached to other attributes, the dosing method and PrEP cost were the two most influential programmatic attributes, explaining over 75·0% of preference variability in the sample. The burden of visits to start (same-day PrEP) accounted for 10.4% of preference variability, while dispensing venue and clinician interaction strategy accounted for ~ 7% each. Additionally, our findings reveal five unique segments (i.e., patient prototypes) among Malaysian MSM with distinct demographic characteristics and preferences for PrEP programs. The segmentation analysis provides a heuristic for creating options for PrEP delivery, given the diversity of MSM. These findings suggest that PrEP uptake in Malaysia can be optimized when there is heterogeneity in the options available in PrEP programs that are ultimately designed around the diverse preferences of key populations. Responsiveness to group-specific preferences in the design of PrEP programs may contribute to increasing PrEP uptake, especially among the least interested segments of the Malaysian MSM^24^.

Most participants in our study reported considerable HIV risk, including inconsistent condom use, multiple anal sex partners, sex with persons living with HIV, and sexualized drug use. Given the efficacy and effectiveness of PrEP, Malaysian MSM must be prioritized for uptake, as they are especially vulnerable to HIV and bear a disproportionate burden of infection. As a high percentage of participants had never tested for HIV and had a recent STI history, PrEP can serve as an entry point to sexual health services and education. Recognizing that HIV risk is not continuous and does not require lifelong therapy, it can be more affordable to pay for PrEP during periods of high risk among MSM. The on-demand dosing, preferred by most respondents in this study, markedly reduces the costs to individuals and society^25^. On-demand PrEP can help MSM maintain privacy by taking it only when needed. This is especially important for partnered MSM (e.g., Group 1) who may not want their partners to know they are taking PrEP^26^. It is also less expensive unless MSM are having condomless sex four or more days per week, potentially adding the appeal to lower cost as well as reduce potential side effects. This dosing method results in higher overall participation rates (92·9% vs. 89·0%) and is favored by the largest segment of participants and the group with the lowest interest in PrEP. These findings highlight the importance of making this dosing method available and increasing the awareness and knowledge of correct dosing for on-demand PrEP to avoid improper use, seroconversion, and antiretroviral resistance^27^. To better support PrEP scale-up in Malaysia, there is a need for guidelines and resources designed to increase providers’ competence in counseling PrEP candidates on multiple dosing strategies that are tailored to their individual preferences^28^.

It is important to note that, when asked explicitly in the cross-sectional survey, most participants (41·2%) preferred injectable PrEP at no cost. This dosing strategy was strongly preferred by groups 1 and 3 in the CBC part of the survey, where participants were at higher risk for HIV and sensitive to PrEP dosing. Our findings are consistent with the literature^29,30^, suggesting that MSM with increased risk for HIV infection (e.g., high perceived HIV risk, history of substance use, and/or condomless sex) were more willing to use injectable PrEP, especially when privacy concerns are elicited. Therefore, as a part of the PrEP scale-up in Malaysia, it is critical to assess the HIV risk of potential clients and recommend injectable PrEP to MSM at high risk for HIV infection. Malaysian providers may need additional training to assess HIV risk, which may be challenging in a context where stigma toward and discrimination against MSM is high^31^. Additionally, the affordability of PrEP services should be a crucial component of all programs in Malaysia, given the cost sensitivity of respondents in our sample. As Malaysia’s Ministry of Health transitions to providing oral PrEP at governmental clinics for free, many of the delivery strategies should be integrated into their plan, including flexible dosing and how clinical and pharmacy services are delivered. Concerns about the potential costs of injectable PrEP were the main obstacle to this dosing strategy, shared by 70·0% of respondents. While being the second most important attribute, the out-of-pocket cost of PrEP is not an insurmountable obstacle to access, given that the majority (~ 80·0%) of respondents are still willing to participate in PrEP programs even though the PrEP is more expensive.

MSM in group 4 had a weak interest in PrEP across all scenarios, suggesting that members of this group are unwilling to use PrEP for reasons unrelated to access. To understand this lack of interest in PrEP, we examined the characteristics of this group. Members of this group had a high prevalence of depression and lower levels of PrEP knowledge. Depression is often associated with lower motivation. These findings also highlight harmful aspects of social stigma that lead to the lack of acceptance by MSM families and friends, causing low self-esteem and poor mental health, resulting in a lack of awareness about HIV prevention and hindering HIV prevention efforts^32^. This is especially true for Group 3, which reported the highest levels of being treated poorly by the healthcare establishment. Appropriate PrEP campaign messaging based on accurate information with the support of community partners will be necessary to reach this group. A successful PrEP program will capitalize on anonymous methods of service (e.g., online consults and courier delivery), offer HIV prevention services in a nonjudgmental manner, and partner with NGOs that already serve the MSM community. Reaching these marginalized subgroups of Malaysian MSM who rarely seek HIV prevention services from mainstream health facilities will require non-traditional delivery platforms, such as peer-to-peer delivery, community-based mobile clinics, pharmacy-led PrEP, and telemedicine and mHealth^33^. The extent to which Malaysia’s Ministry of Health will incorporate these strategies may markedly influence PrEP uptake and HIV prevention efforts.

Our findings suggest that PrEP uptake among Malaysian MSM is most likely to be successful when PrEP implementation is targeted and provided as on-demand with associated education, when high-risk MSM are offered injectable PrEP, and when PrEP is perceived to be affordable. Implementation research is needed to ensure that the provision of injectable PrEP enhances the availability, affordability, and accessibility of HIV prevention services^34^. Uptake can be further increased by providing same-day PrEP initiation and reducing burdensome clinical visits by implementing innovative delivery methods such as pharmacy-led PrEP, mobile community clinics, telemedicine, and mHealth. An effective PrEP program will also emphasize the protection of privacy to maximize uptake. Our findings point to heterogeneity in Malaysian MSM PrEP preferences and suggest that a “one-size-fits-all” strategy would be both insufficient and inefficient as PrEP becomes available for scale-up in the country. A significant share of our sample preferred injectable long-acting or intermittent PrEP to daily oral option. Patient involvement in decisions about their PrEP regimen and delivery options is crucial and needs to be facilitated through a patient decision aid. Similar tools (e.g., insulin initiation) were developed and piloted in Malaysia^35^. A PrEP decision aid, which has been found to be effective in substance-using women^36^, may improve HIV risk perception and facilitate value-congruent choices for Malaysian MSM as well.

There are a few limitations to the study. First, as with other studies using simulated scenarios, stated preferences may not reflect the actual decision-making process in a real-world setting. Future research is needed to evaluate if implementing PrEP based on the study findings results in greater PrEP uptake. Second, the sampling method employed in our study may have led to the possible introduction of sampling bias, which may limit our findings' generalizability. Third, given the sensitive nature of some questions, and despite our efforts to reduce social desirability bias, there is a risk that participants may have felt at times compelled to provide what they thought was the ‘right’ answer. Finally, the survey was implemented in English. While Malaysia is ranked as a high English proficiency country, with over 60% of the population proficient in English, our survey may have limited the participation of MSM who were not fluent in English.

## Conclusion

Our results demonstrate heterogeneity in preferences for PrEP delivery among Malaysian MSM, suggesting the need to maintain a level of choice for different groups of MSM. Differentiated PrEP distribution strategies are needed to provide MSM-friendly and focused services, allowing for PrEP delivery that differs based on dosing, location, provider, and frequency of engagement. On-demand PrEP was a preferred strategy among this sample, while high-risk MSM preferred injectable PrEP. Uptake of PrEP will be successful when it is affordable and offered same day. Providers may need additional training to correctly identify MSM at-risk in the highly stigmatized environment and present PrEP dosing as a continuum of flexible and changeable choices over time. Most importantly, the results highlight the urgency of governmental approval and roll-out of PrEP, given that 85% of our sample had heard of PrEP, over 90% were willing to use it, and at least 30–40% were eligible for PrEP.

## Data Availability

Data can be made available upon reasonable request to the corresponding author.

## References

[1] Vannakit R (2020). Fast-tracking the end of HIV in the Asia Pacific region: Domestic funding of key population-led and civil society organisations. Lancet HIV.

[2] Janamnuaysook R (2021). Demedicalisation of HIV interventions to end HIV in the Asia-Pacific. Sex. Health.

[3] To KW, Lee SS (2018). HIV pre-exposure prophylaxis in South East Asia: A focused review on present situation. Int. J. Infect. Dis..

[4] WHO. *Policy Brief: Pre-exposure Prophylaxis (PrEP): WHO Expands Recommendation on Oral Pre-exposure Prophylaxis of HIV Infection (PrEP)*. (World Health Organization, Switzerland, 2015).

[5] Lau JY, Hung CT, Lee SS (2020). A review of HIV pre-exposure prophylaxis (PrEP) programmes by delivery models in the Asia-Pacific through the healthcare accessibility framework. J. Int. AIDS Soc..

[6] Beres LK (2019). Human-centered design lessons for implementation science: Improving the implementation of a patient-centered care intervention. J. Acquir. Immune Defic. Syndr..

[7] Sharma, M. *et al.* Heterogeneity in individual preferences for HIV testing: A systematic literature review of discrete choice experiments. *EClinicalMedicine* **29–30**, 100653–100653 (2020). 10.1016/j.eclinm.2020.100653 PMC7710637 33305199

[8] MoH. *The Global AIDS Monitoring Report*. (HIV/STI/Hepatitis C Section of Ministry of Health Malaysia, Putrajaya, Malaysia, 2021).

[9] Suleiman A (2015). National Strategic Plan Ending AIDS 2016–2030.

[10] Lim SH (2017). Willingness to use pre-exposure prophylaxis for HIV prevention among men who have sex with men in Malaysia: Findings from an online survey. PLoS ONE.

[11] Eger WH (2022). Exploring drivers of pre-exposure prophylaxis uptake among gay, bisexual, and other men who have sex with men in Malaysia. Int. J. STD & AIDS.

[12] Earnshaw VA (2014). Exploring intentions to discriminate against patients living with HIV/AIDS among future healthcare providers in Malaysia. Trop. Med. Int. Health TM & IH.

[13] Lim, S. H. *et al.* You have to keep yourself hidden: Perspectives from Malaysian Malay-Muslim men who have sex with men on policy, network, community, and individual influences on HIV risk. *J. Homosex.* **67**(1), 104–126 (2020). 10.1080/00918369.2018.1525946 30307803

[14] Earnshaw VA (2016). Stigma toward men who have sex with men among future healthcare providers in Malaysia: Would more interpersonal contact reduce prejudice?. AIDS Behav..

[15] Bourne A (2017). Willingness to use pre-exposure prophylaxis (PrEP) for HIV prevention among men who have sex with men (MSM) in Malaysia: Findings from a qualitative study. J. Int. AIDS Soc..

[16] Beckham SW (2021). Eliciting preferences for HIV prevention technologies: A systematic review. The Patient.

[17] Kroenke K, Spitzer RL, Williams JB (2003). The patient health questionnaire-2: Validity of a two-item depression screener. Med. Care.

[18] McCall H (2015). What is chemsex and why does it matter?. BMJ Br. Med. J..

[19] HRI. *Briefing note: Chemsex and harm reduction for gay men and other men who have sex with men*. (Harm Reduction International, London, 2021).

[20] Shrestha R (2020). Awareness about and willingness to use long-acting injectable pre-exposure prophylaxis (LAI-PrEP) among people who use drugs. J Subst. Abuse Treat.

[21] Dubov A (2018). Optimizing access to PrEP based on MSM preferences: Results of a discrete choice experiment. AIDS Care.

[22] Waschbusch DA (2011). A discrete choice conjoint experiment to evaluate parent preferences for treatment of young, medication naive children with ADHD. J. Clin. Child Adolesc. Psychol..

[23] Orme BK (2006). Getting Started with Conjoint Analysis: Strategies for Product Design and Pricing Research.

[24] Rebe K, Hoosen N, McIntyre JA (2019). Strategies to improve access for MSM in low-income and middle-income countries. Curr. Opin. HIV AIDS.

[25] Ten Brink D (2022). Cost-effectiveness and impact of pre-exposure prophylaxis to prevent HIV among men who have sex with men in Asia: A modelling study. PLoS ONE.

[26] Camp C, Saberi P (2023). Facilitators and barriers of 2–1–1 HIV pre-exposure prophylaxis. PLoS ONE.

[27] Saberi P, Scott HM (2020). On-demand oral pre-exposure prophylaxis with tenofovir/emtricitabine: What every clinician needs to know. J. Gen. Intern. Med..

[28] Haldar P (2021). A rapid review of pre-exposure prophylaxis for HIV in the Asia-Pacific region: Recommendations for scale up and future directions. Sex. Health.

[29] Tolley EE (2020). Acceptability of long-acting injectable cabotegravir (CAB LA) in HIV-uninfected individuals: HPTN 077. AIDS Behav..

[30] Clement ME, Kofron R, Landovitz RJ (2020). Long-acting injectable cabotegravir for the prevention of HIV infection. Curr. Opin. HIV AIDS.

[31] Shrestha R (2020). Using individual stated-preferences to optimize HIV self-testing service delivery among men who have sex with men (MSM) in Malaysia: Results from a conjoint-based analysis. BMC Public Health.

[32] Stall R (2003). Association of co-occurring psychosocial health problems and increased vulnerability to HIV/AIDS among urban men who have sex with men. Am. J. Public Health.

[33] Rousseau E (2021). Novel platforms for biomedical HIV prevention delivery to key populations—community mobile clinics, peer-supported, pharmacy-led PrEP delivery, and the use of telemedicine. Curr. HIV/AIDS Rep..

[34] Schmidt, H.-M.A. *et al.* Long-acting injectable cabotegravir: implementation science needed to advance this additional HIV prevention choice. *J. Int. AIDS Soc.* **25**(7), e25963 (2022). 10.1002/jia2.25963 PMC9334859 35903882

[35] Lee YK, Chirk Jenn N (2017). The state of shared decision making in Malaysia. Z. für Evid. Fortbild. und Qual. im Gesundh..

[36] Meyer J (2022). Preference for and efficacy of a PrEP decision aid for women with substance use disorders. Patient Prefer. Adher..

